# Ca_13_Mab-17, a Novel Anti-Cadherin-13 Monoclonal Antibody for Versatile Applications

**DOI:** 10.3390/antib15030039

**Published:** 2026-05-11

**Authors:** Kai Shimizu, Hiroyuki Suzuki, Mika K. Kaneko, Yukinari Kato

**Affiliations:** Department of Antibody Drug Development, Graduate School of Medicine, Tohoku University, 2-1 Seiryo-machi, Aoba-ku, Sendai 980-8575, Miyagi, Japan; shimizu.kai.p1@dc.tohoku.ac.jp (K.S.); mika.kaneko.d4@tohoku.ac.jp (M.K.K.)

**Keywords:** Cadherin 13, CDH13, monoclonal antibody, Cell-Based Immunization and Screening, flow cytometry, immunohistochemistry

## Abstract

Background/Objectives: Cadherin-13 (CDH13), part of the cadherin family, is attached to the plasma membrane through glycosylphosphatidylinositol. CDH13 plays essential roles in the development of the neurological and vascular systems and is a risk factor for neural and cardiovascular diseases. CDH13 is expressed on the plasma membrane in both mature and uncleaved precursor forms with the prodomain. Although several anti-CDH13 monoclonal antibodies (mAbs) are available for basic research, there have been no reports of anti-CDH13 mAbs that can detect both the mature form and the uncleaved precursor in flow cytometry. Methods: We developed novel anti-human CDH13 mAbs (named Ca_13_Mabs) using the mature form of CDH13-expressed cells as an antigen. Results: Among Ca_13_Mabs, a clone, Ca_13_Mab-17 (IgG_2b_, κ) specifically recognized the mature and uncleaved precursor CDH13-overexpressed Chinese hamster ovary-K1 (CHO/CDH13) cells with no detectable cross-reactivity toward 21 other cadherins by flow cytometry. Ca_13_Mab-17 also detected endogenous CDH13 in human glioblastoma (LN229 and U87MG) and lung mesothelioma (NCI-H2052) cell lines. The dissociation constant (*K*_D_) value of Ca_13_Mab-17 for LN229 was estimated at 4.1 × 10^−8^ M. Furthermore, Ca_13_Mab-17 detected both the mature and uncleaved precursor CDH13 in Western blotting. It also identified new blood vessels and glioblastoma cells by immunohistochemistry. Conclusions: Ca_13_Mab-17 is a versatile tool for detecting both mature and uncleaved precursor forms of CDH13 and has potential for tumor diagnosis and therapy.

## 1. Introduction

Cadherins (CDHs) are crucial for cell–cell adhesion and maintaining normal tissue structure [1]. A large CDH superfamily shares sequence homology with a distinctive domain originally identified in the extracellular regions of CDH1/E-cadherin [2]. The extracellular cadherin (EC) domains contain conserved negatively charged sequences that bind to Ca^2+^ and promote homophilic interactions and cell sorting [3]. Integrating sequence homology with genomic and phylogenetic analyses has enabled the classification of the CDH superfamily into main subgroups [4].

Cadherin-13 (CDH13), also known as truncated-cadherin (T-cadherin) or heart-cadherin (H-cadherin), is a unique glycosylphosphatidylinositol-anchored atypical cadherin [5]. The five EC domains in CDH13 are essential for both homodimerization and interactions with low-density lipoprotein and high-molecular-weight adiponectin in the neurological and vascular systems [6,7]. In the developing nervous system, CDH13-mediated homophilic cell adhesion controls angiogenesis and inhibits neural crest cell migration and motor neuron axon growth [8].

CDH13 exists as approximately a 110 kDa mature form and a 130 kDa uncleaved precursor with the prodomain. Compared to the mature form, the uncleaved precursor exhibits a greater binding affinity for adiponectin [9]. Adiponectin binding increased the cell surface expression and functional activity of 130 kDa CDH13 in endothelial cells, demonstrating positive feedback regulation of CDH13 by adiponectin [9]. CDH13 accumulation in cardiovascular tissues, such as the aorta and heart, allows adiponectin to exert cardioprotective effects [10].

In many tumors, CDH13 is significantly decreased compared to normal tissues and functions as a tumor suppressor [11,12,13]. Lower CDH13 levels have been associated with tumor aggressiveness and poor prognosis in gastric cancer [14,15]. Overexpression of CDH13 induced G0/G1 cell cycle arrest through downregulation of CDK4 and CCND1. Additionally, CDH13 upregulated CDH1/E-cadherin and downregulated vimentin and MMP-2, thereby preventing the migration and invasion of gastric cancer cells [16]. Reduced expression of CDH13 in patients with triple-negative breast cancer is associated with lower postoperative survival, indicating its potential as an independent prognostic marker [17]. Methylation of the CDH13 gene has been reported in not only the above cancers [18,19], but also colorectal cancer [20], ovarian cancer [21], bladder cancer [22], and non-small cell lung cancer [23]. Unlike in other cancers, CDH13 expression is increased in clear cell renal cell carcinomas (ccRCCs). Increased levels of CDH13 are associated with improved overall and progression-free survival in patients with ccRCC, indicating that CDH13 is a novel prognostic biomarker for ccRCC [24].

Monoclonal antibodies (mAbs) that detect CDH13 through Western blotting or immunohistochemistry (IHC) have been developed. However, suitable anti-CDH13 mAbs for flow cytometry have been limited. Using the Cell-Based Immunization and Screening (CBIS) method, our laboratory has developed anti-CDH1 [25] and anti-CDH15 [26] mAbs for flow cytometry, Western blotting, and IHC. The CBIS method involves high-throughput flow cytometry–based screening, and mAbs produced by this method typically recognize conformational epitopes, enabling their use in flow cytometry. Notably, some of these mAbs are also used in Western blotting and IHC. In this study, we employed the CBIS method to develop highly versatile anti-CDH13 mAbs.

## 2. Materials and Methods

### 2.1. Cell Lines

Chinese hamster ovary (CHO)-K1, mouse myeloma P3X63Ag8U.1 (P3U1), human glioblastoma (GBM) LN229, U87MG, and human mesothelioma NCI-H2052 cell lines were obtained from the American Type Culture Collection (ATCC, Manassas, VA, USA). Immortalized normal fibroblast KMST-6 was obtained from the Cell Resource Center for Biomedical Research Institute of Development, Aging, and Cancer at Tohoku University (Sendai, Miyagi, Japan). CHO-K1, P3U1, and CDH-overexpressed CHO-K1 (e.g., CHO/CDH1), NCI-H2052, and KMST-6 were cultured in Roswell Park Memorial Institute (RPMI)-1640 culture medium composed of RPMI-1640 (Nacalai Tesque, Inc., Kyoto, Japan), 10% heat-inactivated fetal bovine serum (FBS, Thermo Fisher Scientific, Inc., Waltham, MA, USA), 100 units/mL penicillin, 100 μg/mL streptomycin, and 0.25 μg/mL amphotericin B (Nacalai Tesque, Inc.). LN229, LN229/CDH13, and U87MG were cultured in Dulbecco’s Modified Eagle Medium (DMEM; Nacalai Tesque, Inc.), supplemented with 10% heat-inactivated FBS (Thermo Fisher Scientific, Inc.), 100 units/mL penicillin, 100 μg/mL streptomycin, and 0.25 μg/mL amphotericin B (Nacalai Tesque, Inc.). Then, cells were maintained in a humidified CO_2_ incubator with 5% CO_2_ and 95% air at 37 °C.

### 2.2. Establishment of Stable Transfectants

Genes encoding human CDH13 (NM_001257) were obtained from the RIKEN BioResource Research Center in Ibaraki, Japan. Full-length CDH13 cDNA was subcloned into the pCAG-Ble vector (FUJIFILM Wako Pure Chemical Corporation, Osaka, Japan). The mature form of CDH13 cDNA lacking the prodomain (amino acids 1 to 138) was subcloned into the pCAG-Ble vector with an N-terminal MAP16 tag. Additionally, the mature form of CDH13 cDNA with an N-terminal PA16 tag was constructed. These plasmids were transfected into LN229 or CHO-K1 cells, and stable transfectants were sorted using an anti-MAP16 tag mAb (clone PMab-1) or an anti-PA16 tag mAb (clone NZ-1) using the Neon transfection system (Thermo Fisher Scientific, Inc.). Finally, MAP16-CDH13-overexpressed LN229 (LN229/CDH13) and PA16-CDH13-overexpressed CHO-K1 (CHO/CDH13) were established.

Type I cadherin-overexpressed CHO-K1: CHO/CDH1, CHO/PA16-CDH2 (CHO/CDH2), CHO/CDH3, CHO/PA16-CDH4 (CHO/CDH4), and CHO/PA16-CDH15 (CHO/CDH15) were previously established [25]. Type II cadherin-overexpressed CHO-K1: CHO/PA16-CDH5 (CHO/CDH5), CHO/CDH6, CHO/PA16-CDH7 (CHO/CDH7), CHO/PA16-CDH8 (CHO/CDH8), CHO/PA16-CDH9 (CHO/CDH9), CHO/PA16-CDH10 (CHO/CDH10), CHO/PA16-CDH11 (CHO/CDH11), CHO/PA16-CDH12 (CHO/CDH12), CHO/PA16-CDH18 (CHO/CDH18), CHO/PA16-CDH19 (CHO/CDH19), CHO/PA16-CDH20 (CHO/CDH20), CHO/PA16-CDH22 (CHO/CDH22), and CHO/PA16-CDH24 (CHO/CDH24) were established previously [27]. Seven-domain (7D) cadherin-overexpressed CHO-K1: CHO/PA16-CDH16 (CHO/CDH16) and CHO/CDH17, and atypical cadherin-overexpressed CHO-K1: CHO/PA16-CDH26 (CHO/CDH26) were previously established [27].

Each cadherin expression was confirmed using an anti-CDH1 mAb (clone Ca_1_Mab-3 [25]), an anti-CDH3 mAb (clone MM0508-9V11, Abcam, Cambridge, UK), an anti-CDH6 mAb (clone 427909, R&D Systems Inc., Minneapolis, MN, USA), an anti-CDH17 mAb (Ca_17_Mab-5, established by our laboratory), and anti-PA16-tag mAb (clone NZ-33 [28]) to detect other cadherins. An anti-CDH13 mAb (clone 392411, MAB3264, rat IgG_2a_) generated by immunization of human CDH13 (Glu23-Ala692) was purchased from R&D Systems, Inc. (Minneapolis, MN, USA).

### 2.3. Hybridoma Production

All animal experiments were approved by the Animal Care and Use Committee of Tohoku University (Permit No. 2022MdA-001) and followed the NIH Guide for the Care and Use of Laboratory Animals. Two 6-week-old female BALB/cAJcl mice, purchased from CLEA Japan (Tokyo, Japan), were intraperitoneally immunized with 1 × 10^8^ cells/mouse of LN229/CDH13. The LN229/CDH13 cells used as immunogen were harvested after brief exposure to 1 mM ethylenediaminetetraacetic acid (EDTA; Nacalai Tesque, Inc.). In the first immunization, Alhydrogel adjuvant 2% (InvivoGen, San Diego, CA, USA) was added. Subsequently, three additional weekly intraperitoneal injections of 1 × 10^8^ LN229/CDH13 cells/mouse were administered without adjuvant. A final booster of 1 × 10^8^ LN229/CDH13 cells/mouse was given intraperitoneally 2 days before harvesting splenocytes from the mice. Cell fusion was performed between the harvested splenocytes from LN229/CDH13-immunized mice and P3U1 cells using polyethylene glycol 1500 (PEG1500; Roche Diagnostics, Indianapolis, IN, USA). Hybridomas were cultured in the RPMI-1640 culture medium supplemented with hypoxanthine, aminopterin, and thymidine (HAT; Thermo Fisher Scientific, Inc.), 5% BriClone (NICB, Dublin, Ireland), and 5 μg/mL of Plasmocin (InvivoGen). The supernatants from the hybridomas were screened by flow cytometry using CHO/CDH13 and parental CHO-K1 cells. The hybridoma supernatant, containing Ca_13_Mab-17 in serum-free medium, was filtered and purified using Ab-Catcher Extra (ProteNova, Higashikagawa, Kagawa, Japan).

### 2.4. Flow Cytometry

Cells were harvested using 1 mM EDTA. Then, they were washed with blocking buffer [0.1% bovine serum albumin in phosphate-buffered saline (PBS)] and incubated with primary mAbs for 30 min at 4 °C. Afterward, cells were stained with Alexa Fluor 488-conjugated anti-mouse or rat IgG (1:2000; Cell Signaling Technology, Inc., Danvers, MA, USA), and fluorescence data were collected using the SA3800 Cell Analyzer (Sony Corp., Tokyo, Japan).

### 2.5. Calculation of the Binding Affinity by Flow Cytometry

Cells were treated with serial dilutions of Ca_13_Mab-17 or 392411. The cells were stained with Alexa Fluor 488-conjugated anti-mouse or rat IgG (1:200 dilution). The dissociation constant (*K*_D_) values of Ca_13_Mab-17 or 392411 were determined using GraphPad Prism 10 software (GraphPad Software, Inc., La Jolla, CA, USA).

### 2.6. Western Blotting

Cell lysates were boiled in sodium dodecyl sulfate (SDS) sample buffer (Nacalai Tesque, Inc.). Proteins (10 µg/lane) were electrophoresed on 5–20% polyacrylamide gels (Wako Pure Chemical Corporation) and transferred onto polyvinylidene difluoride (PVDF) membranes (Merck KGaA, Darmstadt, Germany). After blocking with 4% non-fat milk (Nacalai Tesque, Inc.), PVDF membranes were incubated with 2 μg/mL of Ca_13_Mab-17 and 2 μg/mL of an anti-isocitrate dehydrogenase 1 (IDH1) mAb (clone RcMab-1-mG_1_), followed by incubation with horseradish peroxidase-conjugated anti-mouse IgG (1:1000; Agilent Technologies Inc., Santa Clara, CA, USA). Chemiluminescence signals were developed using Pierce™ ECL Plus (Thermo Fisher Scientific, Inc.) or ImmunoStar LD (Wako Pure Chemical Corporation). The signals were imaged with ChemiDoc Touch MP (Bio-Rad Laboratories, Inc., Berkeley, CA, USA).

### 2.7. IHC Using Cell Blocks and Tissue Microarrays

All procedures of IHC were performed using VENTANA BenchMark ULTRA PLUS (Roche Diagnostics, Indianapolis, IN, USA). Cells were fixed with 4% paraformaldehyde, and the cell blocks were prepared using iPGell (Genostaff Co., Ltd., Tokyo, Japan). The formalin-fixed paraffin-embedded (FFPE) cell sections were stained with Ca_13_Mab-17 (0.2 or 2 μg/mL) or RdMab-20 (2 μg/mL, IgG_2b_ isotype control). Tissue microarrays (GBM GL806e and colorectal cancer CO483b, US Biomax Inc., Rockville, MD, USA) were stained with Ca_13_Mab-17 (2 μg/mL) or LpMab-12 (an anti-podoplanin mAb, 2 μg/mL). The detection was conducted using the VENTANA BenchMark ULTRA PLUS with ultraView Universal DAB Detection Kit (Roche Diagnostics).

## 3. Results

### 3.1. Development of Anti-CDH13 mAbs by the CBIS Method

**Figure 1:**
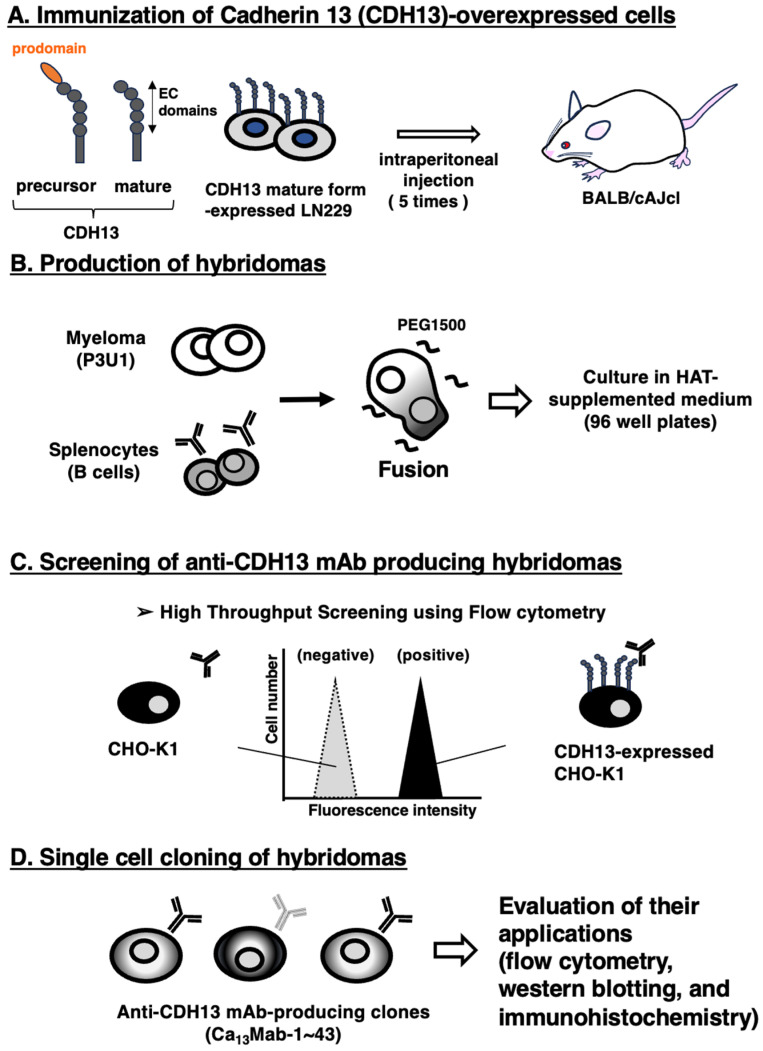
Procedure of anti-CDH13 mAbs production. (**A**) Structure of precursor and mature form of CDH13. The mature form CDH13-expressed LN229 (LN229/CDH13) was immunized in BALB/cAJcl mice. (**B**) After five immunizations, splenocytes were fused with P3U1. (**C**) The supernatants from hybridomas were screened using CHO-K1 and CHO/CDH13 by flow cytometry. (**D**) Ca_13_Mabs, anti-CDH13 mAb-producing hybridoma clones, were established through limiting dilution.

To obtain anti-CDH13 mAbs that react with both the mature and uncleaved precursor forms of CDH13, we prepared an immunogen, LN229/CDH13, which expresses mature CDH13 as described in the Section 2.2 and Figure 1A. The LN229/CDH13 cells (1 × 10^8^ cells/mouse) were intraperitoneally injected five times into two BALB/cAJcl mice (Figure 1A). Hybridomas were generated by fusing splenocytes with myeloma P3U1 cells (Figure 1B). Supernatants from the hybridomas were screened to identify those positive for CHO/CDH13 and negative for CHO-K1 (Figure 1C). Limiting dilution was then performed, and a total of 43 clones producing anti-CDH13 mAbs were established. The supernatants were further evaluated for their utility in flow cytometry, Western blotting, and immunohistochemistry (Figure 1D). Ultimately, Ca_13_Mab-17 (IgG_2b_, κ) was selected because it can be used in all applications (http://www.med-tohoku-antibody.com/topics/001_paper_antibody_PDIS.htm, accessed on 8 May 2026).

### 3.2. Flow Cytometric Analysis of Ca_13_Mab-17 Against CHO-K1 and CHO/CDH13

**Figure 2:**
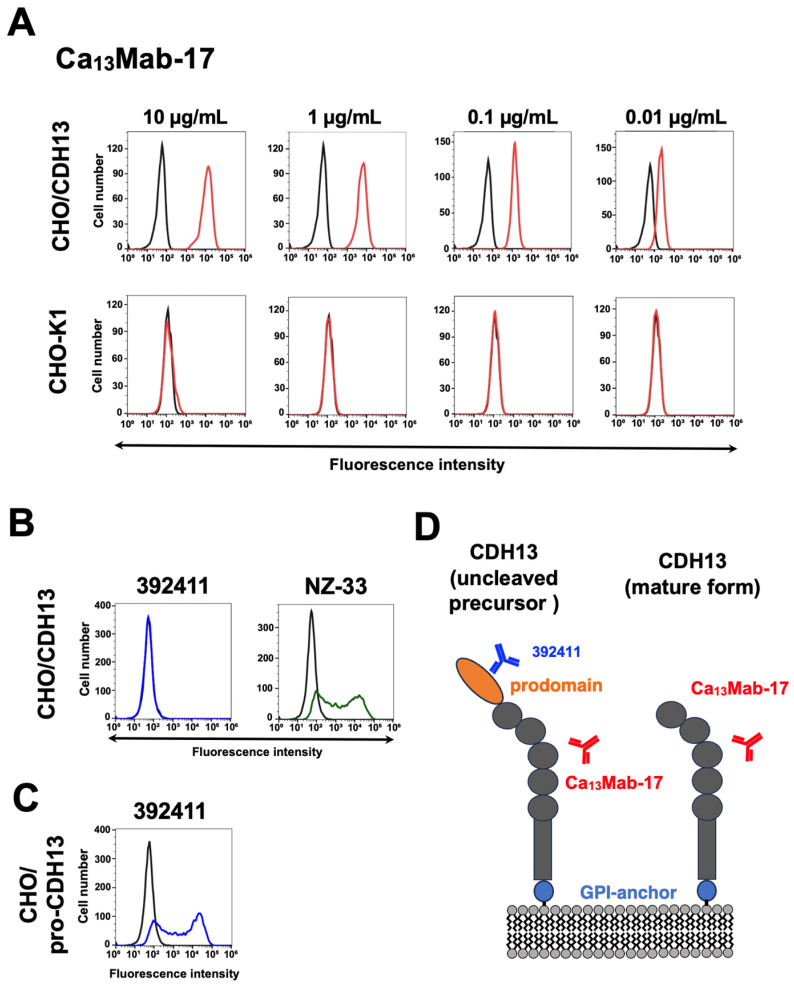
Flow cytometric analysis of Ca_13_Mab-17. (**A**) CHO-K1 and CHO/CDH13 were treated with Ca_13_Mab-17 at the indicated concentrations (red) or with blocking buffer (black, negative control). The Ca_13_Mab-17-treated cells were incubated with Alexa Fluor 488-conjugated anti-mouse IgG. (**B**) CHO-K1 transiently transfected with PA16-CDH13 was treated with 2 µg/mL of 392411 (bule) or 1 µg/mL of an anti-PA tag mAb, NZ-33 (green). The cells were incubated with Alexa Fluor 488-conjugated anti-rat IgG or anti-mouse IgG, respectively. (**C**) CHO-K1 transiently transfected with prodomain-containing CDH13 (pro-CDH13) was treated with 2 µg/mL of 392411 (bule). The cells were incubated with Alexa Fluor 488-conjugated anti-rat IgG. Fluorescence data were collected using the SA3800 Cell Analyzer. (**D**) Schematic representation of the Ca_13_Mab-17 and 392411 recognitions.

Figure 2A shows the flow cytometric analysis using purified Ca_13_Mab-17 against CHO/CDH13 and CHO-K1. Ca_13_Mab-17 reacted in a dose-dependent manner with CHO/CDH13 from 10 to 0.01 μg/mL. In contrast, Ca_13_Mab-17 did not recognize CHO-K1 even at 10 μg/mL. Additionally, a commercially available anti-CDH13 mAb (clone 392411) did not recognize CHO-K1 transfected with mature form of CDH13 (Figure 2B). In contrast, 392411 recognized prodomain-containing CDH13-transfected CHO-K1 (Figure 2C). These results suggest that Ca_13_Mab-17 recognizes both uncleaved precursor and mature form of CDH13, while 392411 recognizes uncleaved precursor of CDH13 (Figure 2D).

### 3.3. Determination of the Specificity of Ca_13_Mab-17 Using CDHs-Overexpressed CHO-K1

**Figure 3:**
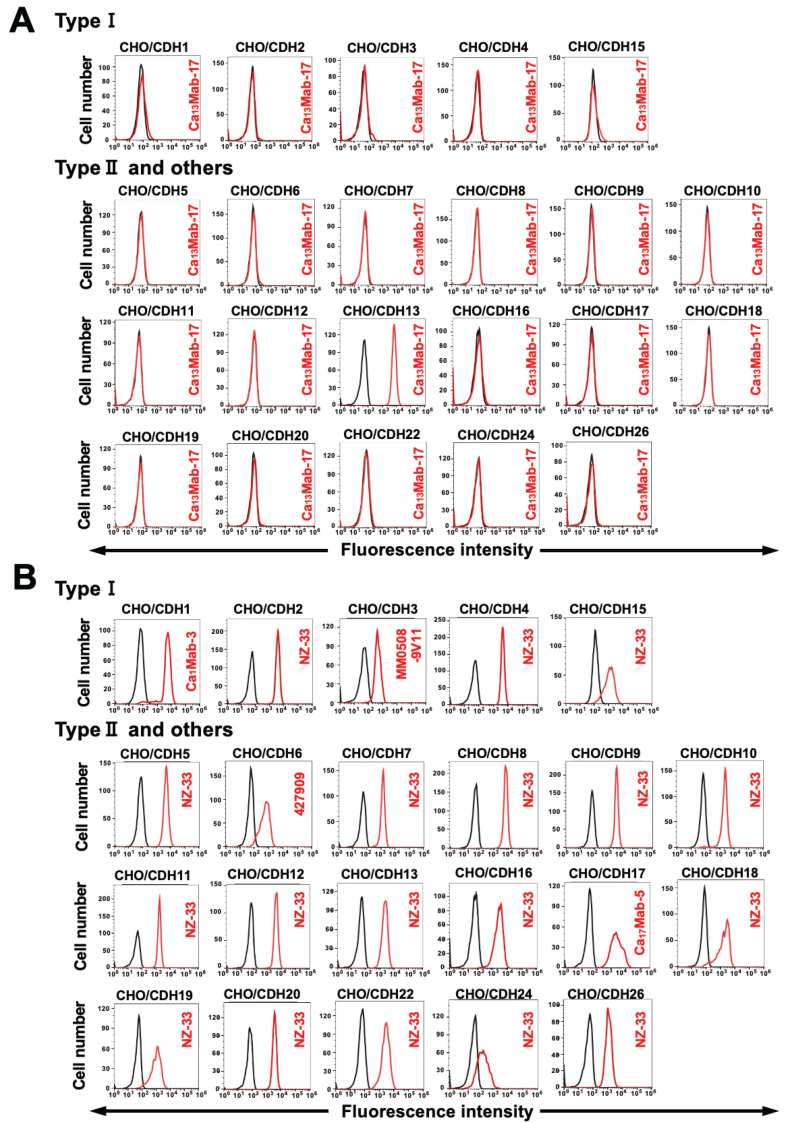
Specificity of Ca_13_Mab-17. (**A**) The type I cadherins (CDH1, CDH2, CDH3, CDH4, and CDH15), type II cadherins (CDH5, CDH6, CDH7, CDH8, CDH9, CDH10, CDH11, CDH12, CDH18, CDH13, CDH20, CDH22, and CDH24), a truncated cadherin (CDH13), 7D cadherins (CDH16 and CDH17), and an atypical cadherin (CDH26)-overexpressed CHO-K1 were treated with 10 µg/mL of Ca_13_Mab-17 (red) or with control blocking buffer (black, negative control), followed by treatment with anti-mouse IgG conjugated with Alexa Fluor 488. (**B**) Each cadherin expression was confirmed by 1 µg/mL of an anti-CDH1 mAb (clone Ca_1_Mab-3), 1 µg/mL of an anti-CDH3 mAb (clone MM0508-9V11), 1 µg/mL of an anti-CDH6 mAb (clone 427909), 1 µg/mL of an anti-CDH17 mAb (clone Ca_17_Mab-5), and 0.1 µg/mL of an anti-PA16-tag mAb (clone NZ-33) to detect other CDHs, followed by the treatment with Alexa Fluor 488-conjugated secondary mAbs. The fluorescence data were collected using the SA3800 Cell Analyzer.

We previously established CHO-K1 cells, which overexpressed type I cadherins (CHO/CDH1, CHO/CDH2, CHO/CDH3, CHO/CDH4, and CHO/CDH15) [25,26], type II cadherins (CHO/CDH5, CHO/CDH6, CHO/CDH7, CHO/CDH8, CHO/CDH9, CHO/CDH10, CHO/CDH11, CHO/CDH12, CHO/CDH18, CHO/CDH19, CHO/CDH20, CHO/CDH22, and CHO/CDH24), 7D cadherins (CHO/CDH16 and CHO/CDH17), and an atypical cadherin (CHO/CDH26) [27]. Therefore, the specificity of Ca_13_Mab-17 to those cadherins was determined. As shown in Figure 3A, Ca_13_Mab-17 recognized CHO/CDH13 but did not react with other cadherins-overexpressed CHO-K1. The cell surface expression of each cadherin was confirmed in Figure 3B. These results indicate that Ca_13_Mab-17 is a specific mAb to CDH13 among those CDHs.

### 3.4. Flow Cytometric Analysis of Ca_13_Mab-17 and 392411 Against Endogenous CDH13-Positive Cells

**Figure 4:**
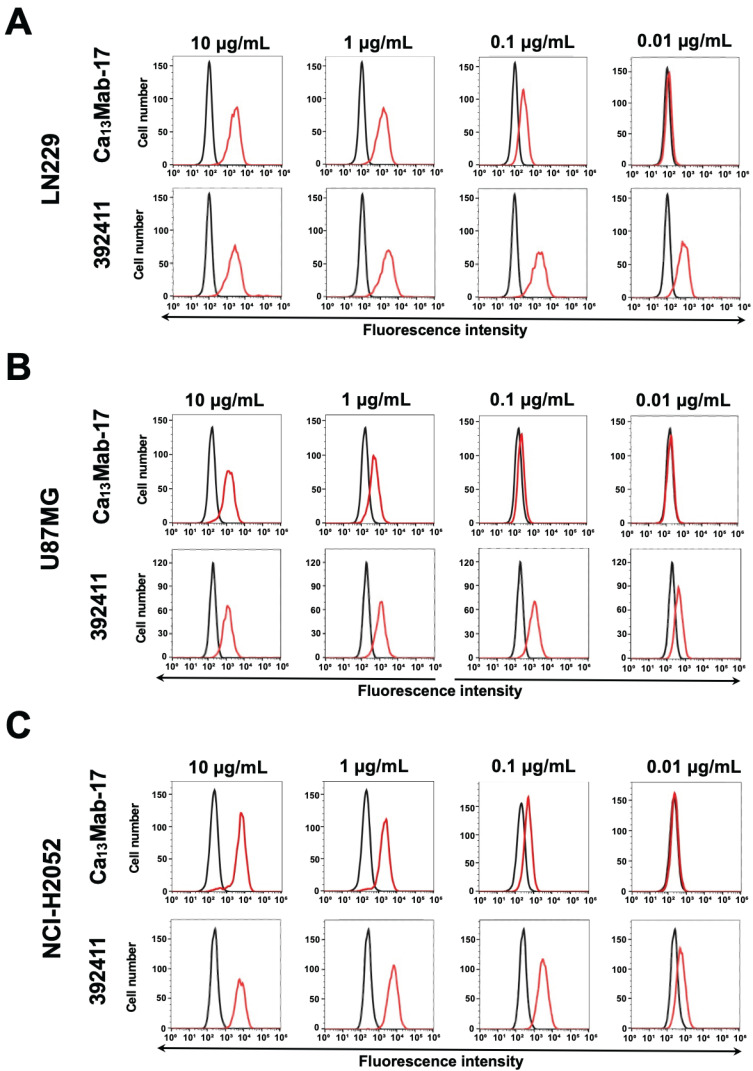
Flow cytometric analysis of Ca_13_Mab-17 for detection of endogenous CDH13. LN229 (**A**), U87MG (**B**), and NCI-H2052 (**C**) were treated with Ca_13_Mab-17 at the indicated concentrations (red) or with blocking buffer (black, negative control). The Ca_13_Mab-17-treated cells were incubated with Alexa Fluor 488-conjugated anti-mouse IgG. Fluorescence data were collected using the SA3800 Cell Analyzer.

We then screened the cell lines that react to Ca_13_Mab-17 and 392411. As shown in Figure 4, both Ca_13_Mab-17 and 392411 recognized endogenous CDH13 in LN229 (Figure 4A), U87MG (Figure 4B), and NCI-H2052 (Figure 4C) in a dose-dependent manner. At high concentrations (10 and 1 μg/mL), Ca_13_Mab-17 exhibited similar reactivity to 392411. At lower concentrations (0.1 and 0.01 μg/mL), 392411 showed higher reactivity. These results indicate that both Ca_13_Mab-17 and 392411 can detect endogenous CDH13 in flow cytometry.

**Figure 5:**
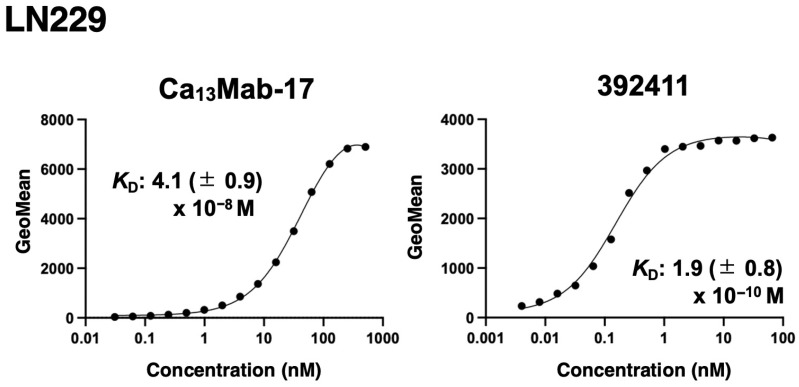
Determination of binding affinity of Ca_13_Mab-17 by flow cytometry. LN229 was suspended in 100 µL of serially diluted Ca_13_Mab-17. Then, cells were reacted with Alexa Fluor 488-conjugated anti-mouse IgG. Subsequently, the geometric mean fluorescence values were obtained using the SA3800 Cell Analyzer. The average *K*_D_ values (± standard deviation) from three independent measurements were calculated by GraphPad PRISM 10 software. The representative graphs were shown.

The binding affinity of Ca_13_Mab-17 and 392411 was measured with LN229 using flow cytometry. The *K*_D_ values for Ca_13_Mab-17 and 392411 with LN229 were 4.1 (±0.9) × 10^−8^ M and 1.9 (±0.8) × 10^−10^ M, respectively (Figure 5). These results show that Ca_13_Mab-17 has moderate affinity for endogenous CDH13.

### 3.5. Determination of the Specificity of Ca_13_Mab-17 Using CDHs-Overexpressed CHO-K1

**Figure 6:**
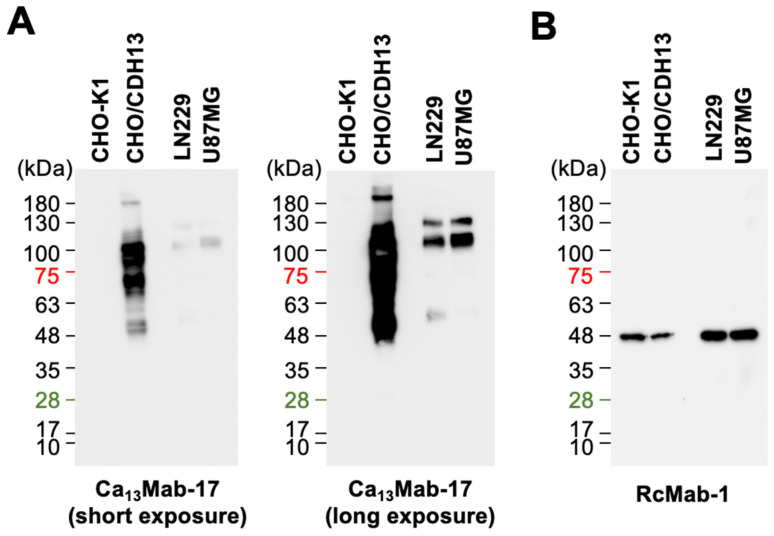
Western blotting using Ca_13_Mab-17. Cell lysates (10 μg/lane) from CHO-K1, CHO/CDH13, LN229, and U87MG were electrophoresed and transferred to polyvinylidene difluoride membranes. The membranes were incubated with 1 μg/mL of Ca_13_Mab-17 (**A**) or 1 μg/mL of RcMab-1 (an anti-IDH1 mAb) (**B**), followed by the treatment with anti-mouse or anti-rat IgG-conjugated with horseradish peroxidase for Ca_13_Mab-17 or RcMab-1, respectively.

We tested whether Ca_13_Mab-17 can be used in Western blotting. As shown in Figure 6, Ca_13_Mab-17 detected CDH13 as the main band around 100 kDa in CHO/CDH13 cell lysates, while no band appeared in parental CHO-K1 cells. Additionally, Ca_13_Mab-17 detected endogenous CDH13 in LN229 and U87MG cells at 110 and 130 kDa (Figure 6A). An anti-IDH1 mAb (clone RcMab-1) was used as an internal control (Figure 6B). These results demonstrate that Ca_13_Mab-17 can identify CDH13 in Western blotting.

### 3.6. IHC Using Ca_13_Mab-17 in Formalin-Fixed Paraffin-Embedded Cell Blocks and Tissue Microarrays

**Figure 7:**
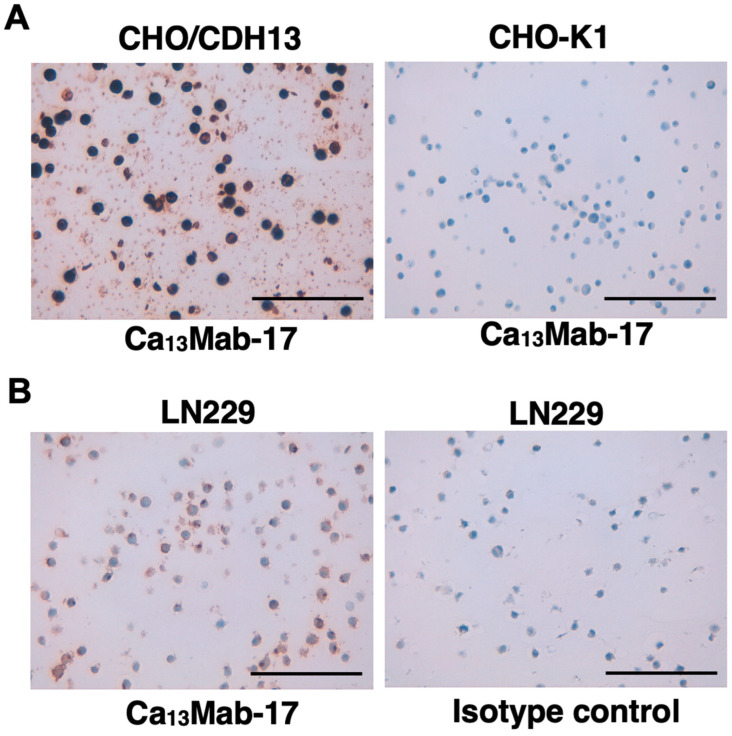
Immunohistochemistry using Ca_13_Mab-17 in formalin-fixed paraffin-embedded cell blocks. (**A**) CHO/CDH13 and CHO-K1 sections were treated with 0.2 μg/mL of Ca_13_Mab-17. (**B**) LN229 sections were treated with 2 μg/mL of Ca_13_Mab-17 or 2 μg/mL of RdMab-20 (IgG_2b_ isotype control). The staining was performed using VENTANA BenchMark ULTRA PLUS with the ultraView Universal DAB Detection Kit. Scale bar = 100 μm.

We tested whether Ca_13_Mab-17 is suitable for IHC in FFPE sections from CHO-K1 and CHO/CDH13. Ca_13_Mab-17 showed intense membranous and cytoplasmic staining in CHO/CDH13 but not in CHO-K1 (Figure 7A). Furthermore, Ca_13_Mab-17 also displayed membranous staining in LN229, but the isotype control mAb (RdMab-20) did not (Figure 7B). These results indicate that Ca_13_Mab-17 can detect both exogenous and endogenous CDH13 in IHC of FFPE sections from cultured cells.

**Figure 8:**
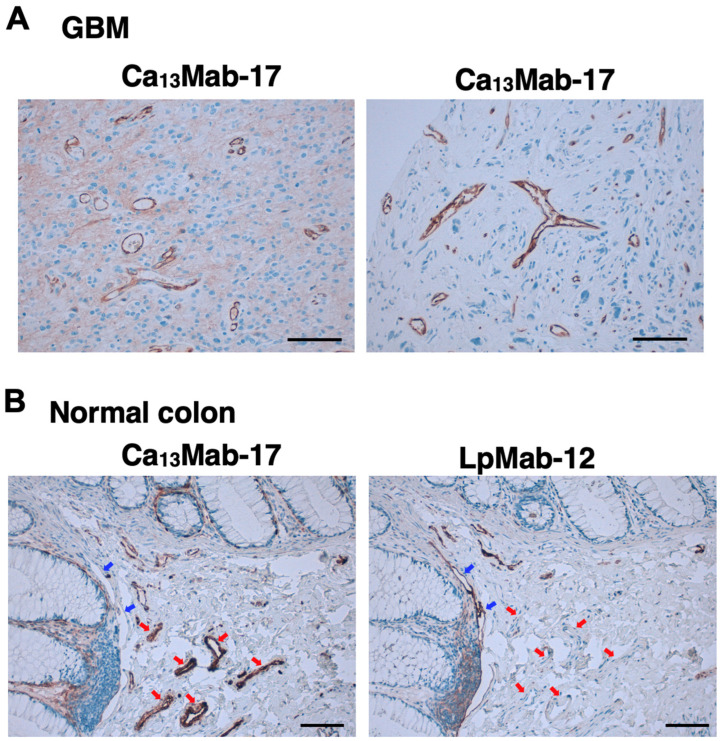
Immunohistochemistry using Ca_13_Mab-17 in tissue arrays. (**A**) The GBM tissue array (GL806e) was treated with 2 μg/mL of Ca_13_Mab-17. The representative spots were shown. (**B**) Sequential sections of the colorectal cancer tissue array (CO483b) were treated with 2 μg/mL of Ca_13_Mab-17 or 2 μg/mL of LpMab-12 (an anti-podoplanin mAb) to detect lymphatic endothelial cells. A normal colon spot was shown. Red arrows indicate CDH13-positive endothelial cells. Blue arrows indicate the podoplanin-positive lymphatic endothelial cells. The staining was performed using VENTANA BenchMark ULTRA PLUS with the ultraView Universal DAB Detection Kit. Scale bar = 100 μm.

We next stained a GBM tissue microarray. As shown in Figure 8A, Ca_13_Mab-17 exhibited strong staining of tubular structures and moderate staining of GBM tissue (Figure 8A, left). Cases with tubular structure positivity and GBM negativity were also observed (Figure 8A, right). The results are summarized in Table 1. Typical images and scores were presented in Supplementary Figure S1. We then investigated whether Ca_13_Mab-17 specifically detects vascular endothelial cells. We stained serial sections of a colorectal cancer microarray using Ca_13_Mab-17 or LpMab-12, an anti-podoplanin mAb used to detect lymphatic endothelial cells. Although Ca_13_Mab-17 did not stain colorectal cancers, Ca_13_Mab-17 preferentially stained blood vessels that were podoplanin-negative (Figure 8B, red arrows). Conversely, Ca_13_Mab-17 did not stain large lymphatic vessels that were podoplanin-positive (Figure 8B, blue arrows).

## 4. Discussion

In this study, we developed novel anti-CDH13 mAbs by immunizing with the mature form of CDH13-overexpressed LN229 (Figure 1). A clone Ca_13_Mab-17 recognized both exogenous and endogenous CDH13 in flow cytometry (Figure 2 and Figure 4) and IHC (Figure 7 and Figure 8). Importantly, Ca_13_Mab-17 showed specificity for CDH13 without detectable cross-reactivity to other 21 cadherins, including types I, II, 7D, and other CDH molecules (Figure 3). Therefore, Ca_13_Mab-17 could be useful for the specific isolation of CDH13-positive cells via fluorescence-activated cell sorting. In Western blotting, Ca_13_Mab-17 detected both the 110 and 130 kDa forms of endogenous CDH13 in LN229 and U87MG (Figure 6), indicating that Ca_13_Mab-17 can recognize both the mature form and the uncleaved precursor of CDH13. Identifying the Ca_13_Mab-17 epitope is essential for investigating its neutralization activity and developing more specific anti-CDH13 mAbs in the future.

A commercially available anti-CDH13 mAb (clone 392411) recognized endogenous CDH13-expressing cancer cells with higher reactivity and affinity compared to Ca_13_Mab-17 (Figure 4 and Figure 5), but did not recognize CHO/CDH13 (Figure 2B) in flow cytometry. 392411 was developed by immunization with human CDH13 (Glu23-Ala692) containing the prodomain. We confirmed that 392411 recognizes prodomain-containing CDH13 (Figure 2C). Although we performed Western blotting using 392411 to detect the uncleaved precursor form of CDH13, we could not detect it in LN229 and U87MG lysates. According to the manufacturer’s home page (https://www.rndsystems.com/products/human-mouse-cadherin-13-antibody-392411_mab3264, accessed on 8 May 2026), 392411 showed no cross-reactivity with recombinant human CDH1, CDH2, CDH3, CDH5, CDH8, CDH11, CDH12, or CDH17 in enzyme-linked immunosorbent assay and Western blotting. These results indicate that 392411 can detect the uncleaved precursor of CDH13 with high reactivity and affinity.

Ca_13_Mab-17 recognized the human GBM and lung mesothelioma cell lines in flow cytometry (Figure 4). CDH13 expression increases during primary astrocyte physiological growth arrest in response to contact inhibition [29]. In C6 glioma cell lines, CDH13 expression leads to growth suppression by upregulating CDKN1A, indicating a tumor suppressor role in glioma cells [29]. However, the role of CDH13 in specific tumor types remains controversial, and the relationship between its expression and function is highly context-dependent. In prostate cancer, CDH13 exhibits stage-dependent effects, promoting differentiation and chemotherapy sensitivity in early stages but gradually diminishing during disease progression [13]. Moreover, elevated levels of CDH13 expression have been observed in hepatocellular carcinoma [30] and osteosarcoma [31]. These findings suggest that the relationship between the expression pattern and function of CDH13 depends on tumor stage and type. Ca_13_Mab-17 helps analyze the molecular role of CDH13 and potential therapies for CDH13-positive tumors.

We previously cloned cDNAs from hybridomas and produced recombinant mouse IgG_2a_-type mAbs to enhance antibody-dependent cellular cytotoxicity (ADCC). We also evaluated antitumor activity using human tumor xenograft models [32,33]. We have cloned the cDNA of Ca_13_Mab-17, and the subclass-changed IgG_2a_-type Ca_13_Mab-17 will be produced and evaluated for antitumor activities using in vitro ADCC assay and tumor xenograft models.

Ca_13_Mab-17 is suitable for IHC of cell specimens (Figure 7) and tissue microarrays (Figure 8). Notably, IHC was performed on an automated slide-staining system, which ensures consistent and reproducible staining conditions. Ca_13_Mab-17 detected CDH13 in both tumor tissue and vascular structures in GBM (Figure 8A) and the lamina propria of the colon (Figure 8B). CDH13 has been reported to be detected in endothelial cells, smooth muscle cells, and pericytes [34]. In tumor angiogenesis, the vessels exhibit an aberrant distribution of the basement membrane, marked differences in capillary size, and partially dissociated pericytes and smooth muscle cells from endothelial cells and the basement membrane [35,36]. GBM exhibits aggressive angiogenesis with no lymphatic vessels or lymphangiogenesis [37]. Therefore, Ca_13_Mab-17 can detect CDH13-positive vascular capillaries in GBM. In the lamina propria of the colon, Ca_13_Mab-17 did not stain in podoplanin-positive large lymphatic vessels (Figure 8B). Many endothelial markers such as CD31, CD34, and CDH5/VE-cadherin are detected in both vascular and lymphatic endothelial cells [38]. Ca_13_Mab-17 could distinguish vascular endothelial cells from lymphatic endothelial cells. Further double-staining analyses are essential to prove the exclusive staining of CDH13 and podoplanin in vascular and lymphatic endothelial cells, respectively.

## Figures and Tables

**Table 1:** Immunohistochemistry of GBM microarray (GL806e) by Ca_13_Mab-17.

					Ca_13_Mab-17 Staining	
No	Age	Sex	Organ/Anatomic Site	Pathology Diagnosis	Tumor	Blood Vessel
1	17	M	Cerebrum/right frontal lobe	Epithelioid glioblastoma	-	1+
2	59	F	Cerebrum/right frontal lobe	Glioblastoma multiforme	2+	-
3	59	M	Cerebrum/occipital lobe	Glioblastoma multiforme	2+	-
4	10	F	Cerebrum/right frontal lobe	Glioblastoma	-	3+
5	33	F	Cerebrum/Left temporal lobe	Epithelioid glioblastoma	-	2+
6	52	M	Cerebrum/left frontal lobe	Glioblastoma multiforme	-	2+
7	27	F	Cerebrum/left frontal lobe	Epithelioid glioblastoma	-	-
8	42	M	Cerebrum/left parietal lobe	Glioblastoma multiforme	-	-
9	30	M	Cerebrum	Glioblastoma	-	-
10	32	M	Cerebrum	Glioblastoma multiforme	-	2+
11	25	M	Cerebrum/right parietal lobe	Epithelioid glioblastoma	2+	-
12	22	M	Cerebrum/right parietal lobe	Glioblastoma	-	2+
13	20	M	Cerebrum/right temporal lobe	Glioblastoma multiforme	2+	-
14	56	M	Cerebrum/left parietal lobe	Epithelioid glioblastoma	1+	-
15	76	F	Cerebrum/occipital lobe	Glioblastoma with necrosis	2+	3+
16	59	F	Cerebrum/left frontal lobe	Epithelioid glioblastoma	-	3+
17	42	M	Cerebrum/occipital lobe	Glioblastoma	1+	2+
18	56	M	Cerebrum	Epithelioid glioblastoma	1+	3+
19	41	M	Cerebrum/Left temporal lobe	Glioblastoma	1+	-
20	57	M	Cerebrum	Glioblastoma multiforme	2+	-
21	48	F	Cerebrum/left frontal lobe	Glioblastoma	2+	-
22	21	M	Cerebrum/Left temporal lobe	Gliosarcoma	-	3+
23	25	M	Cerebrum/parietal lobe	Glioblastoma	-	3+
24	38	M	Cerebrum/left frontal lobe	Glioblastoma multiforme	-	2+
25	40	M	Cerebrum/right temporal lobe	Glioblastoma multiforme	-	-
26	8	F	Cerebrum/left frontal lobe	Epithelioid glioblastoma	-	-
27	38	F	Cerebrum/left frontal lobe	Glioblastoma	2+	3+
28	15	M	Cerebrum	Giant cell glioblastoma multiforme	-	2+
29	58	M	Cerebrum/occipital lobe	Glioblastoma	-	2+
30	16	F	Cerebrum	Glioblastoma	1+	-
31	37	M	Cerebrum/right temporal lobe	Glioblastoma	1+	-
32	80	M	Cerebrum/left frontal lobe	Glioblastoma	-	2+
33	31	M	Cerebrum/left parietal lobe	Glioblastoma	-	1+
34	44	M	Cerebrum/right temporal lobe	Glioblastoma multiforme	2+	-
35	27	F	Cerebrum/left frontal lobe	Glioblastoma multiforme	-	3+
36	32	M	Cerebrum	Cerebral tissue (Normal)	3+	-
37	38	F	Cerebrum	Cerebral tissue (Normal)	3+	-
38	37	M	Cerebrum	Cerebral tissue (Normal)	3+	-
39			Cerebrum	Cerebral tissue (Normal)	3+	-
40	26	M	Cerebrum	Cerebral tissue (Normal)	3+	-

-, No stain; 1+, Weak intensity; 2+, Moderate intensity; 3+, Strong intensity.

## Data Availability

The data presented in this study are available in the article.

## Supplementary Materials

The following supporting information can be downloaded at: https://www.mdpi.com/article/10.3390/antib15030039/s1, Figure S1: Immunohistochemistry using Ca_13_Mab-17.
