# Supplementary material for: Ca13Mab-17, a Novel Anti-Cadherin-13 Monoclonal Antibody for Versatile Applications
Source: Antibodies (Basel). 2026 May 11;15(3):39. doi: 10.3390/antib15030039 (PMC13214481; doi:10.3390/antib15030039)
Supplement: Supplementary file 1 [file antibodies-15-00039-s001.zip › antibodies-4251526-supplementary.pdf]

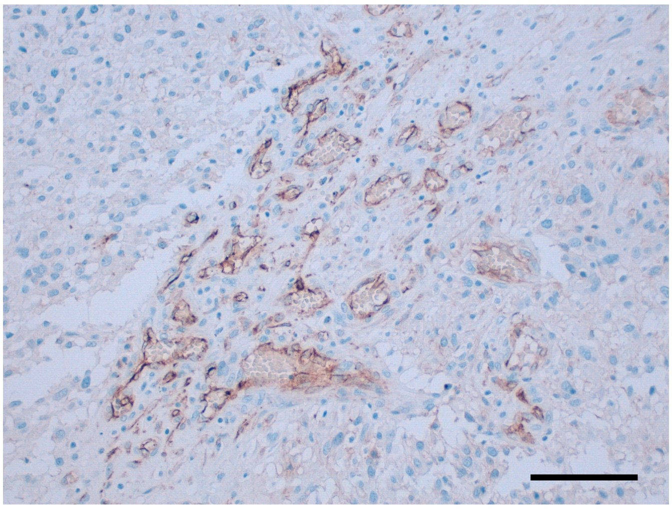

(Tumor 1+, blood vessel 3+)

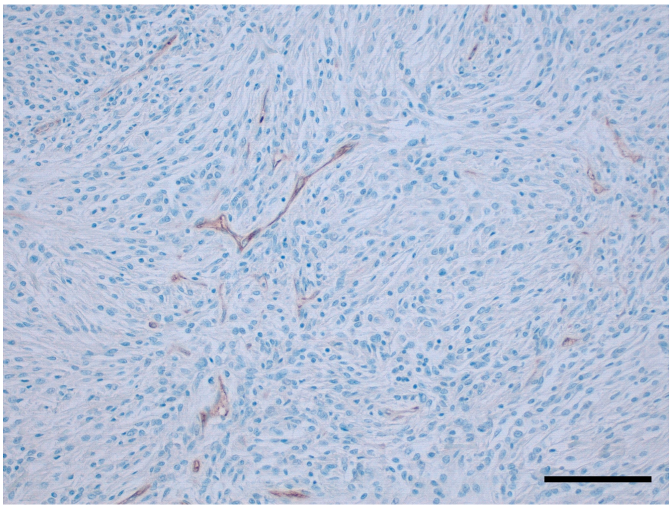

(Tumor -, blood vessel 2+)

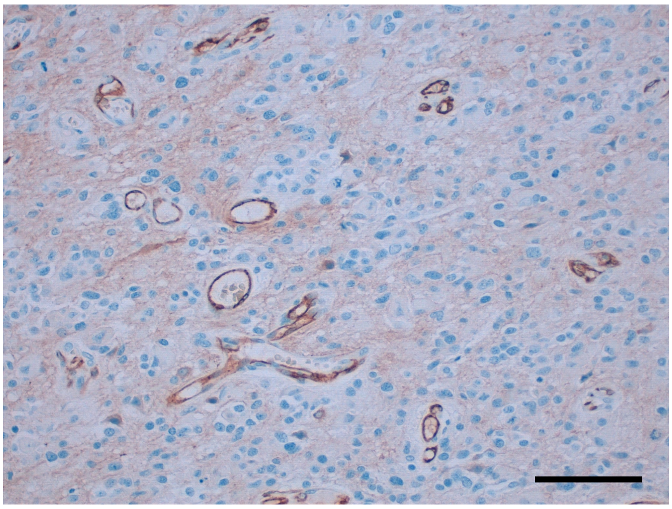

(Tumor 2+, blood vessel 3+, Fig. 8A)

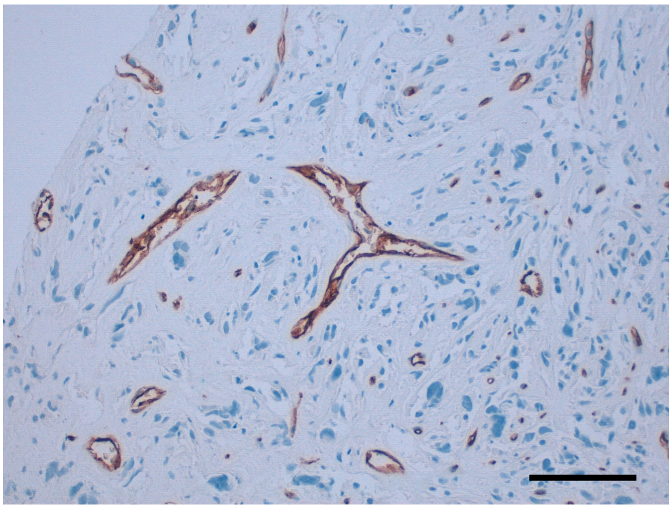

(Tumor -, blood vessel 3 +, Fig. 8A)

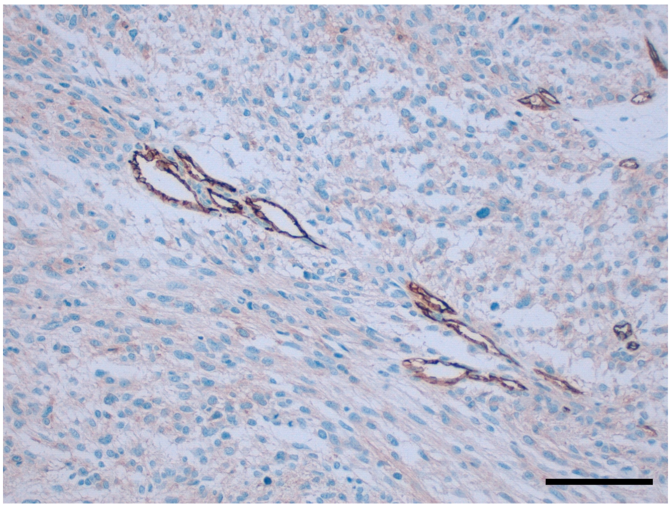

(Tumor 2+, blood vessel 3+)

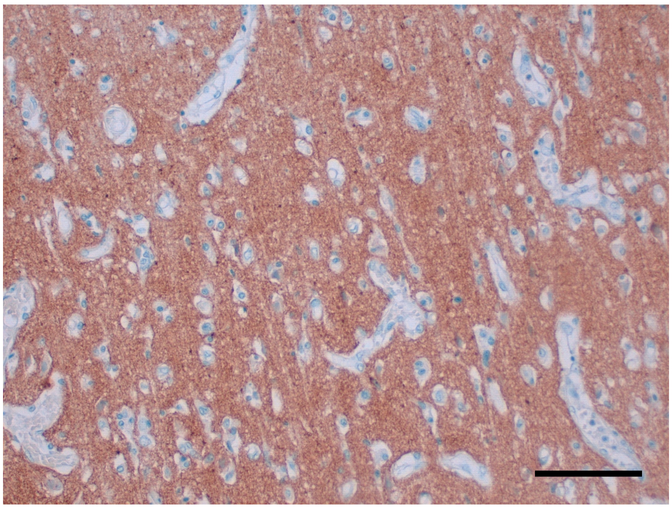

(Normal cerebral tissue 3+, blood vessel -)

Supplementary Figure S1. Immunohistochemistry using Ca_{13}Mab-17. The GBM tissue array (GL806e) was treated with 2 μg/mL of Ca_{13}Mab-17. Images and scores were shown. The staining was performed using VENTANA BenchMark ULTRA PLUS with the ultraView Universal DAB Detection Kit. Scale bar = 100 μm.
